# Early Onset of Tenofovir-Related Fanconi Syndrome in a Child with Acute Hepatitis B: A Case Report and Systematic Review of Literature

**DOI:** 10.1155/2017/3921027

**Published:** 2017-11-15

**Authors:** Renato Pascale, Viola Guardigni, Lorenzo Badia, Francesca Volpato, Pierluigi Viale, Gabriella Verucchi

**Affiliations:** ^1^Infectious Diseases Unit, Department of Medical and Surgical Science, S. Orsola-Malpighi Hospital, University of Bologna, Bologna, Italy; ^2^Research Centre for the Study of Hepatitis, University of Bologna, Bologna, Italy

## Abstract

Tenofovir disoproxil fumarate- (TDF-) related nephropathy is known to be a long-term complication of this drug, more commonly observed in HIV-infected patients, but occurring also in hepatitis B. Cases of Fanconi Syndrome associated with TDF have been reported in adult patients, usually as a long-term complication of chronic hepatitis B treatment. We present here a case of a 12-year-old male developing a severe acute HBV hepatitis treated with TDF. The patient achieved an early virological and biochemical response, but with a subsequent onset of proximal renal tubular damage, consistent with Fanconi Syndrome. After withdrawing this drug and switching to Entecavir, a complete resolution of tubulopathy and, after 6 months, a complete HBsAg seroconversion occurred. To our knowledge, this is the first report of an early renal injury due to TDF-therapy in a pediatric patient treated for acute hepatitis B.

## 1. Introduction

In 1992, World Health Organization set the inclusion of hepatitis B vaccine into their childhood vaccination programs as a goal for all countries worldwide [1]. Indeed, in the United States, acute hepatitis B cases from the National Notifiable Disease Surveillance System showed an overall low incidence rate of 0.9/100,000 population in 2011 [2]. Similar trend was reported in Italy, with an incidence of 0.85/100,000 population, in 2012 [3]. Severe acute hepatitis B is defined by coagulopathy (INR > 1.5) or a protracted course (i.e., persistent symptoms or marked jaundice for >4 weeks) or signs of acute liver failure, representing a risk for an incipient fulminant hepatitis. European Association For The Study Of The Liver and European Society of Pediatric Gastroenterology suggest that both pediatric and adult patients with this condition might benefit from treatment with Nucleoside Analogue (NA) in order to prevent development of fulminant hepatitis [4, 5].

Reports mainly described lamivudine (LAM) therapy even if, in many experts' opinion, Entecavir (ETV) or Tenofovir disoproxil fumarate (TDF) should be used [5, 6], since these third-generation NAs have higher antiviral potency and are less likely to induce resistance than LAM [4, 7, 8]. Moreover, a case of fulminant hepatitis B in a 4-month-old infant successfully treated with Tenofovir was reported [9].

We present here the first case of an early-onset renal injury, consistent with Fanconi Syndrome, during TDF-therapy in a child treated for acute hepatitis B. Furthermore, we report a systematic review of scientific literature on this topic.

## 2. Methods

We conducted a literature search using PubMed, PubMed Central, and Medline databases, reviewing all the case reports published in English. Search terms included “Tenofovir”, “Fanconi Syndrome”, “Renal injury”, “Hepatits B”, and “children”. The initial search was performed in January 2015 and repeated for new references in May 2017.

## 3. Case Report

A 12-year-old male affected by an osteosarcoma of femur, diagnosed in August 2012, after several neoadjuvant cycles of chemotherapy with adriamycin, cisplatin, ifosfamide, and methotrexate, underwent a surgical removal of femur in December 2012. After surgery, additional chemotherapy cycles were performed, and, during the last one (in June 2013), an unexpected hypertransaminasemia (ALT: 456 U/L) was detected. Further laboratory tests confirmed ALT and AST alterations progressively worsening (AST: 280 UI/ml and ALT: 620 UI/ml). The serologic panel showed acute HBV infection (HBsAg positive, HBcIgM positive, HBeAg negative, and HBeAb positive, HBV-DNA: >170.000.000 UI/ml), even though it was shown that the child was correctly vaccinated for HBV, as his parents and relatives were. D genotype was identified. Considering that HBsAg was negative at presurgical screening (while HBsAb was not tested), a diagnosis of acute HBV infection in a patient nonresponder to vaccination was formulated, hypothesizing an infection occurred during his surgical or oncological course. Despite moderate elevation in aminotransferases and INR (1.40) we decided to start an off-label treatment with Tenofovir considering a substantial risk for evolution toward a fulminant hepatitis B (which is more likely in childhood than in other age ranges [6]). The dosage assessed by age and body weight was 245 mg once a day. After 30 days of therapy, HBV-DNA had decreased of 4log⁡(10), HBsAg was declining, and transaminases and coagulation parameters were normalized; no signs of acute encephalopathy developed at any time. Considering patient's oncological history and the planned further chemotherapy cycles, we decided to maintain NA-treatment until HBsAg clearance. Nevertheless, after 5 months of TDF-treatment, normoglycemic glycosuria (glycemia 88 mg/dl, glycosuria 70 mg/dl), phosphaturia with hypophosphatemia (reduced tubular absorption of phosphorus (56%), phosphatemia 1.9 mg/dl, proteinuria (albuminuria 100 mg/dl at dipstick, proteinuria: 0.2 g/24 h), glomerular filtration rate (GFR) 70 ml/min/1.73 m^2^, and urine creatinine 0.47 g/day were detected, leading to diagnosis of Fanconi Syndrome associated with TDF-therapy.

**Figure 1:**
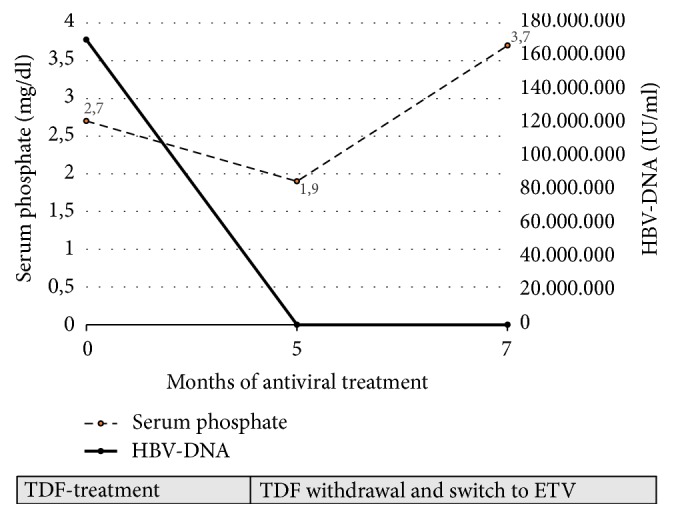
Serum phosphate and HBV-DNA levels over Tenofovir treatment and after its withdrawal.

Assuming that tubular damage was possibly related to TDF (enhancing a preexisting renal impairment secondary to chemotherapy), the latter was withdrawn and a concomitant switch to ETV was decided. At that time, there were no indications for pediatric use of ETV but, considering reports of safety and efficacy in pediatric population treated for chronic HBV infection, a dosage of 0.015 mg/kg/day was prescribed. ETV therapy was well tolerated and maintained HBV-DNA negativity. Indeed, after 2 months of this treatment a complete resolution of tubulopathy was observed and after 6 months a complete HBs seroconversion (HBsAg negative; HBsAb: 15 UI/ml) occurred (Figure 1). Currently, the oncological disease is in remission and NA therapy has been stopped after 18 months from seroconversion. Persistence of HBs seroconversion has been confirmed after 2 years from the end of treatment.

## 4. Discussion

Fanconi Syndrome, characterized by normoglycemic glycosuria, hypophosphatemia, aminoaciduria, proteinuria, metabolic acidosis, and hypouricemia, may be caused by NtRTIs (like TDF) [16] and is widely reported in HIV-infected patients, also pediatric. Probably, in this special population, the direct role of HIV damaging kidney and concomitant use of other drugs, such as protease inhibitor, are closely related to renal impairment [17–19].

Renal injury due to TDF monotherapy in hepatitis B infection is less common, although several studies have reported chronic tubular damage and reduction in eGFR in patients treated with this antiviral [4] and Tenofovir alafenamide (TAF) has been now demonstrated to be safer than TDF in registrational trials [4, 6]. To date, there are few published reports of TDF-related Fanconi syndrome (Table 1): all of them have been described in adult patients with CHB. To the best of our knowledge, this is the first report of an early-onset TDF-related renal injury in a child with acute hepatitis B.

Majority of patients described in literature [10–15, 20] developed tubular complications after long-term TDF-administration (after an average of 24 months) for hepatitis B, suggesting that Fanconi Syndrome is a late onset complication of this drug. Our case presents some peculiarities: early onset of the tubulopathy (reported only by Samarkos et al. [14] and Hwang et al. [15]), young age of the patients, and TDF-administration for acute HBV-related hepatitis. In our patient chemotherapy could have played an important role in enhancing kidney damage, since he had been recently treated with ifosfamide and cisplatin, which are drugs with a well-known nephrotoxicity [16]. This relevant predisposing factor, not characterizing the other patients reported in literature, might explain such an early onset of TDF-related toxicity.

After the switch to Entecavir, tubular function progressively improved and HBV-DNA continued to be undetectable. At the time of decision, ETV was not yet registered for pediatric use, while safety and effectiveness of ETV in treating children with CHB are now proved [8, 10] and the drug is registered for use in this population. Acute HBV symptomatic infection is rare in pediatric age (due to vaccination), and it can vary from a mild to a fulminant hepatitis. Classic symptoms are present in 30–50% of older children and adolescents with acute hepatitis B and include fever, jaundice, nausea and vomiting, abdominal pain, liver tenderness, and fatigue, which last approximately 2-3 months [4, 5]. In our report, the patient had only laboratory tests alteration (increase in transaminases and coagulation parameters), without clinical findings.

HBV vaccination has an excellent record of safety and effectiveness (response in over 90% of the immune-competent individuals) [21]. However, a substantial rate of nonresponse to vaccination may occur. Almost 1–10% of healthy individuals fail to generate a protective antibody response to hepatitis B vaccine (more frequent in healthy adults compared to neonates) [22]; many factors associated with a nonresponse to HBV vaccination have been described, including incorrect administration of the vaccine, impaired vaccine storage conditions, drug abuse, smoking, genetic factors, obesity, chronic kidney disease, celiac disease, thalassemia, type I diabetes mellitus, and Down's Syndrome [21–23]. We considered our patient as nonresponder to hepatitis B vaccination but we do not know how the child became infected with HBV: it might be speculated that it happened during his many hospitalizations. Indeed, though unexpected, cases of transmission of HBV in health-care setting have been reported also in recent years [24].

We decided to start NA therapy considering the severity of illness, with risk of acute hepatic failure development. The potential need of long-term therapy with risk to develop resistance, induced us to prefer a third-generation NA rather than lamivudine [4, 7, 8]. Our first choice was TDF, which is known to have higher antiviral potency and to induce a lower rate of resistance than lamivudine [7]. At the time of the decision there were already many data supporting the use of TDF in children and adolescents suffering of chronic hepatitis B (CHB), as well as part of antiretroviral therapy in HIV-infected children [10, 19, 25]. Particularly, in the study of Murray et al. TDF was shown to be highly effective and safe for HBV suppression in 52 adolescents with CHB observed for 72 weeks [10]. An anecdotal case of fulminant hepatitis B of a 4-month-old infant successfully treated with Tenofovir was also reported [9].

In conclusion, despite the presence of an effective vaccine, new HBV infections are still possible (also in health-care settings) and testing HBV markers should be mandatory in every patient exposed to immunosuppressive treatment, regardless of vaccination report. Last generation NAs (e.g., TDF, ETV) are effective not only in CHB but also in acute hepatitis B.

Our case highlights the potential risks for TDF-related renal injury and raises some concerns about using this antiviral in frail patients with concomitant risk factors for acute tubular damage, such as chemotherapy. Therefore, a close monitoring of renal and tubular function in patients undergoing TDF-treatment should be performed in any case, and the choice of NA for hepatitis B should be based on patients' characteristics, considering the high virological efficacy of all the available antivirals.

## Figures and Tables

**Table 1:** Review of cases of TDF-associated Fanconi Syndrome during Hepatitis B therapy.

	Age	Sex	TDF-treatment duration	Risk factor	Serum creatinine (mg/dl)	eGFR^*∗*^ (ml/min)	Serum phosphate (mg/dl)	Serum bicarbonate (mmol/L)	Uric Acid (mg/dl)	Phosphate fractional excretion (%)	Glycosuria° (mg/dl)	Proteinuria (g/24 h)
Murray et al. [10]	39	M	24 months	Adefovir exposure	1.44	59	1.86	n/a	3.5	n/a	yes	0.6
Murray et al. [10]	54	M	24 months	Hypertension	1.53	51	2.1	n/a	1.34	elevated	n/a	0.2
Magalhães-Costa et al. [11]	82	M	6 months	Adefovir exposure, diabetes	n/a	n/a	1.1	13.9	1.7	65.8%	yes	yes
Gracey et al. [12]	58	M	42 months	Adefovir exposure	1.32	55	2.1	19.3	n/a	n/a	500	0.5
Gracey et al. [12]	62	M	45 months	Hypertension	3.35	18	1.7	19.5	n/a	n/a	400	n/a
Viganò et al. [13]	44	F	3 months	Diabetes	3.22	20	2.6	11	2.5	elevated^∧^	n/a	yes
Samarkos et al. [14]	58	M	12 months	Adefovir exposure, hypertension	1.45	n/a	1.4	17.1	n/a	elevated^∧∧^	500	0.96
Hwang et al. [15]	40	M	36 months	No	1.5	58.6	1.3	n/a	1.9	41%	200	0.3
Our case	12	M	5 months	Ifosfamide, Cisplatin	0.47	70	1.9	n/a	n/a	56%	70	0.2

^*∗*^By MDRD formula; °by dipstick. ^∧^Tubular maximal transport of phosphate reabsorption to the glomerular filtration rate transport (TmP/GFR): 0.008 mg/dl; ^∧∧^TmP/GFR: 0.66 mmol/L.
